# Malignant high-grade unclassifiable spindle cell sarcoma in the hand: A diagnostic and therapeutic challenge

**DOI:** 10.1016/j.jpra.2025.08.001

**Published:** 2025-08-09

**Authors:** Victor Hugo Garzón-Ortega, Kenzo Alejandro Fukumoto-Inukai, Carlos A. Morales-Morales, Yusef Jiménez-Murat

**Affiliations:** a Department of Plastic and Reconstructive Surgery, Hospital General “Dr. Manuel Gea Gonzalez” Calzada de Tlalpan 4800, Belisario Domínguez Section 16, 14080 Mexico City, Mexico; b Chief of the Hand Clinic of Plastic and Reconstructive Surgery, Hospital General “Dr. Manuel Gea González”, Calzada de Tlalpan 4800, Belisario Domínguez Section 16, 14080 Mexico City, Mexico

**Keywords:** Hand, Spindle cell sarcoma, High-grade sarcoma, Soft tissue tumor, Surgical resection Functional preservation, Case report

## Abstract

Introduction: Spindle cell sarcoma is a rare malignancy characterized by spindle-shaped cells, often mimicking other tumors and posing diagnostic challenges due to its nonspecific clinical features. This case highlights the importance of a multidisciplinary approach and demonstrates the feasibility of complete tumor resection with functional preservation, even in large and complex tumors.

Case Presentation: A 51-year-old woman with no significant medical history presented with a large, exophytic mass on her left hand, causing notable functional impairment in daily activities. Physical examination revealed a rubbery mass, measuring 15 × 8 cm. Imaging studies, including radiography, computed tomography, and magnetic resonance imaging, were performed. Given the impact of grasp functionality on daily life, surgical resection was scheduled. The excised tissue was subsequently submitted for histopathological evaluation.

Results: Imaging demonstrated a vascularized mass without bone involvement. Initial biopsy suggested multicentric cellular dermatofibroma. However, histopathological examination of the excised specimen revealed findings consistent with high-grade spindle cell sarcoma. Immunohistochemistry showed positivity for CD99 and CD56, with negative markers for S-100, factor XIII, TLE-1, CD34, SOX10, H3K27ME, SS18 and actin. At the 10-month follow-up, she remained recurrence-free with preserved hand function.

Conclusions: A meticulous surgical approach is vital for the management of the balance of complete tumor excision and preservation of hand function. A thorough histopathological examination is necessary for diagnosis. Collaboration across disciplines is key to improving treatment outcomes.

## Introduction

Spindle cell sarcoma (ICD-O-3: 8801/3)^1^ is a rare undifferentiated sarcoma, that shares histopathological characteristics with other sarcomas, spindle cell sarcoma (SCS) is an exclusive diagnosis.^2^ The upper limb is the most commonly affected site, the estimated annual incidence is 5/1,000,000/year.^3^ Surgical management has demonstrated a positive impact on survival, compared to radiotherapy management.^4^^,^^5^

## Objective

This case report evaluates the surgical management of a high-grade spindle cell sarcoma (HGSCC) in the hand, focusing on resection techniques, functional preservation, and multidisciplinary collaboration

## Patient information and clinical findings

**Figure 1:**
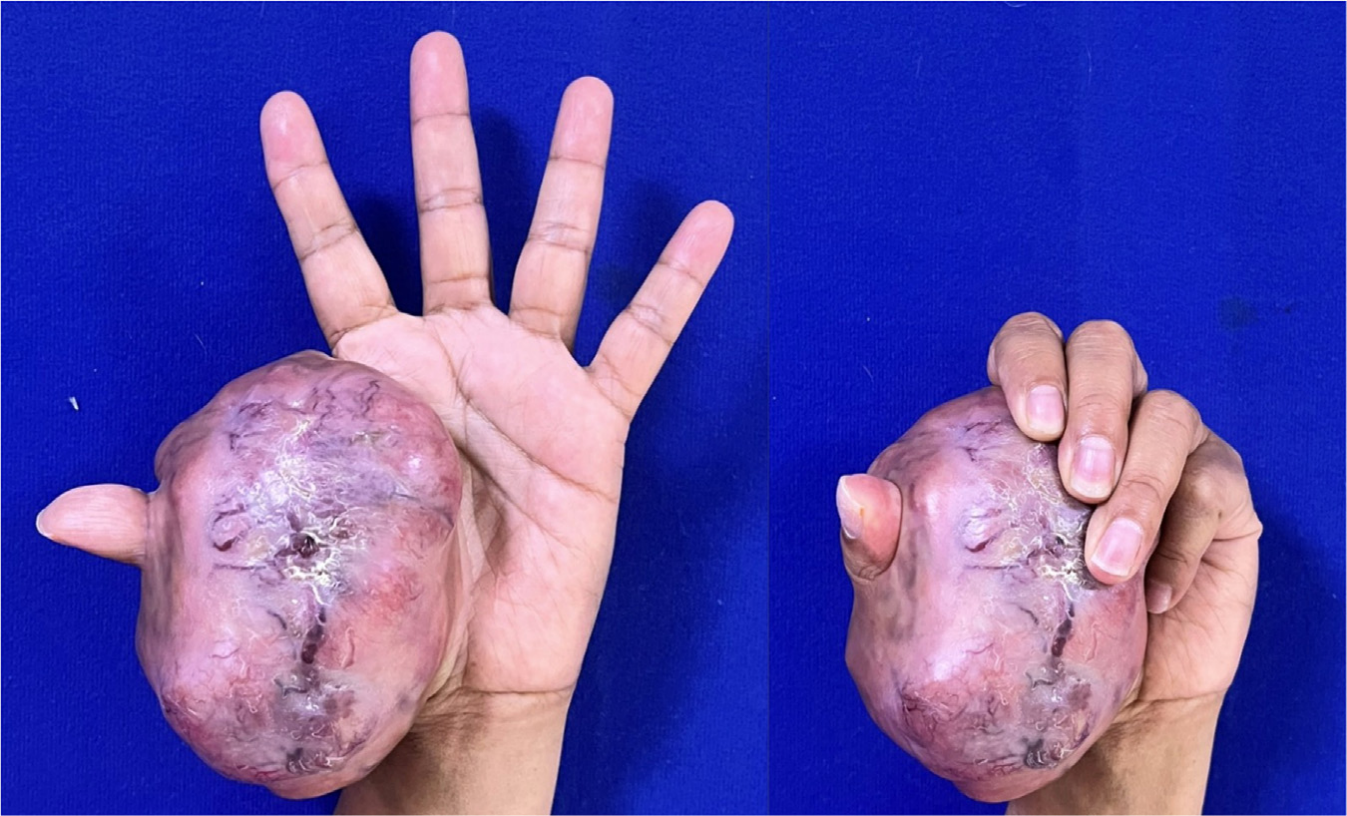
Preoperative clinical image showing a large tumor involving the first digit and metacarpal region. The lesion is extensive, preventing the patient from flexing the thumb.

A previously healthy 51-year-old caucasian female domestic worker with a mass on her left hand, measuring 15 × 8 cm extending from the radial border of the thumb to the palmar aspect of the second and third fingers of the left hand (Figure 1).

## Timeline

The patient noticed an insidious mass that began to grow around June 2022, it does not develop at a constant size rate, also without a homogeneous growth pattern, in October 2023 an excisional biopsy was performed with a pathology diagnosis of dermatofibroma. Towards January 2024, the mass accelerated the growth rate, so she decided to come to our service in April 2024 due to significant impairment of motor skills. She did not seek help earlier due to her work, in addition, the poor level of education affects the time of diagnosis and management.

## Diagnostic assessment and interpretation

**Figure 2:**
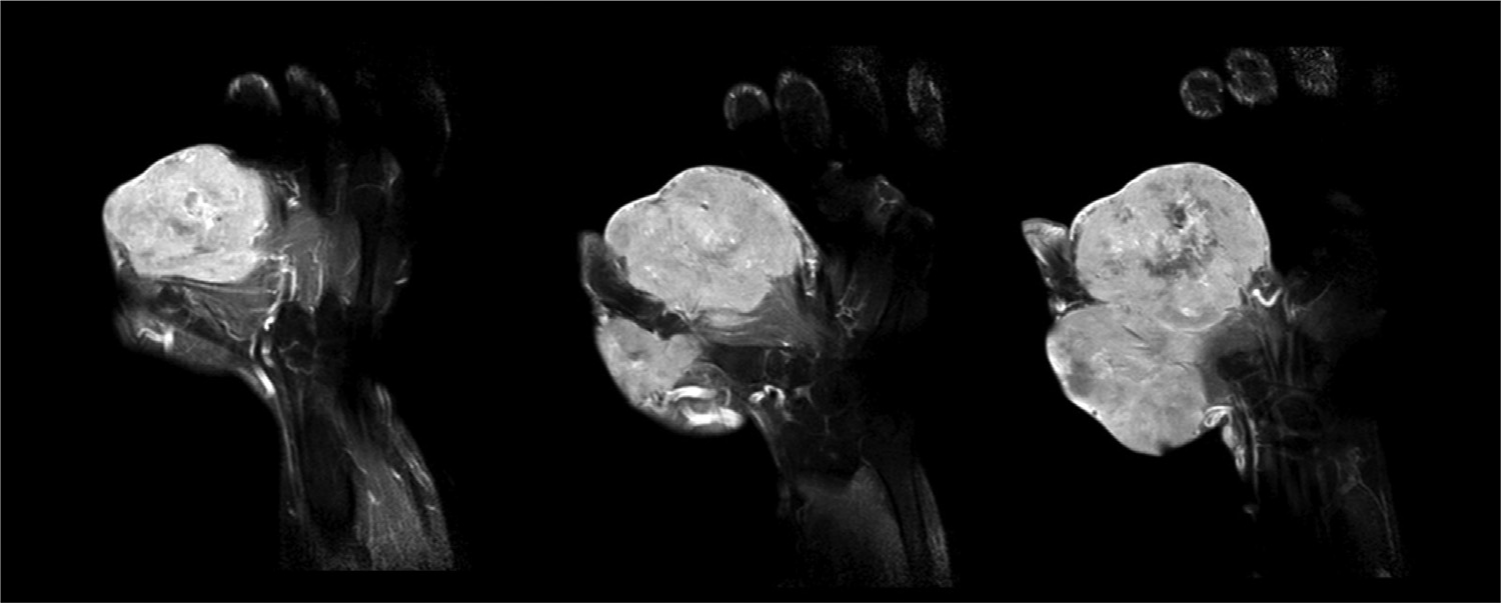
Axial T2-weighted MRI demonstrating tortuous vasculature involving the radial and ulnar digital arteries of the thumb (RDDAT, UDDAT) and the first dorsal metacarpal artery (FDMA).

Imaging studies, including radiographs, and MRI (Figure 2), revealed a soft tissue mass without bone involvement. MRI (T2-weighted) demonstrated tortuous vasculature involving the radial and ulnar digital arteries of the thumb (RDDAT, UDDAT) and the first dorsal metacarpal artery (FDMA). Given the absence of other complexities, en bloc resection via a dorsal approach was planned.

## Intervention

**Figure 3:**
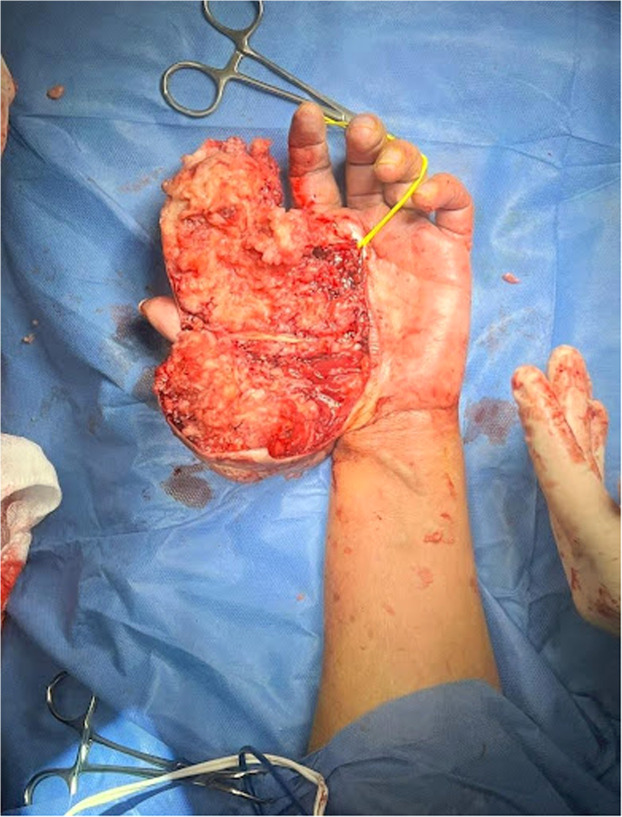
Intraoperative view showing a central butterfly-like incision over the mass, facilitating exposure and excision.

Under tourniquet control, a dorsal incision was made, preserving the first interdigital space. The tumor appeared encapsulated with fibroid and fatty characteristics. Due to the tumor's vascular complexity, the incision was modified to a central approach (Figure 3) allowing complete resection with free margins while preserving tendons and neurovascular structures. A full-thickness skin graft was used for closure, and the limb was splinted postoperatively.

## Follow-up and outcomes

**Figure 4:**
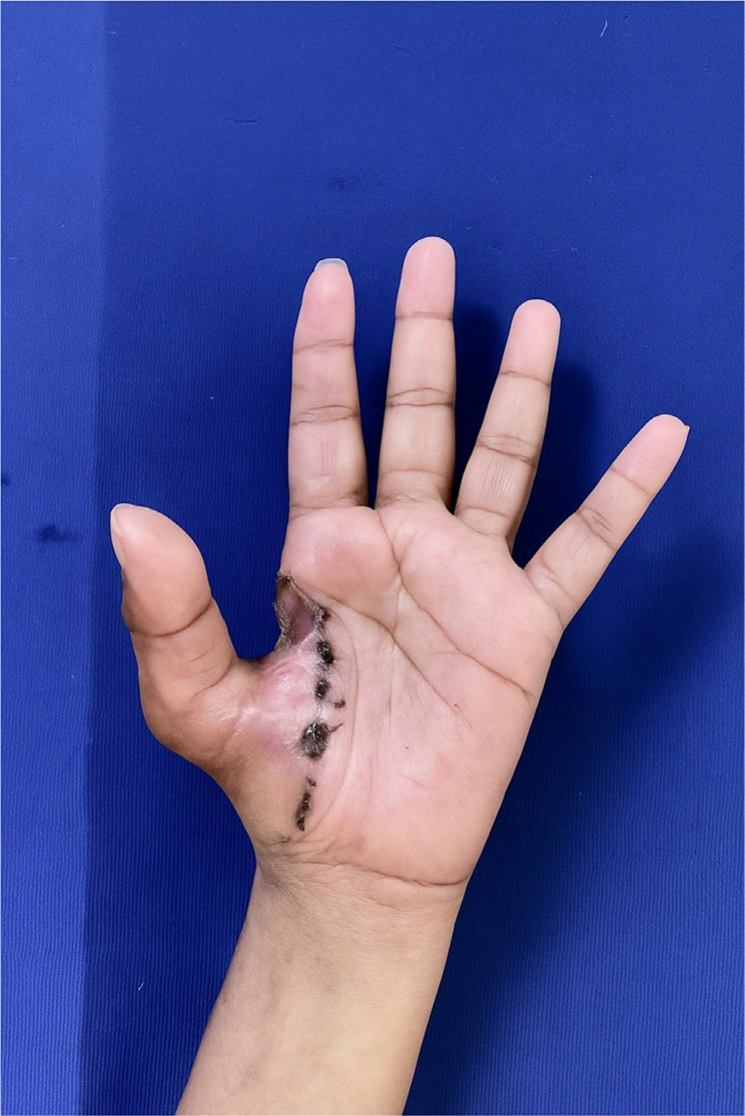
3 month follow-up.

We successfully performed a function-preserving resection of an HGSCC of the hand, achieving complete tumor excision while minimizing damage to surrounding structures. The patient underwent oncological follow-up and rehabilitation. At the 4-week evaluation, there was significant improvement in strength and range of motion, allowing a return to daily activities. Histopathological analysis, including immunohistochemistry, confirmed the diagnosis, with positivity for CD99 and CD56, and tumor-free margins. The 3 month image (Figure 4) shows stable wound healing with no signs of recurrence, supporting the feasibility of our function-sparing approach.

At the 10-month follow-up, the patient remains recurrence-free, with preserved hand function and motor strength graded M4 against gravity. This case highlights the feasibility of achieving oncological control while maintaining hand functionality, even in complex tumor resections.

## Discussion

SCS are rare malignant tumors. Their presentation doesn't have a defined behavior, it varies depending on the zone and growth index. Predominantly observed in men (1.4:1 men to women).^3^ It is important to maintain a low diagnostic suspicion even when the tumor does not correspond to previously defined histopathological diagnosis, we should always do immunohistochemistry studies. We recommend always assuming these masses are malign and plan accordingly, multidisciplinary management with an oncological approach is a must.

Differential diagnoses that we should take into account are giant dermatofibroma, synovial sarcoma, and dermatofibrosarcoma.^2^^,^^6^ Traditionally, en bloc resection with wide margins has been the standard of care. However, given the critical functional importance of the hand, and particularly the thumb, we carefully considered a function-sparing approach in this case. While non-en bloc resection is generally discouraged due to concerns about cell seeding, we hypothesized that meticulous surgical technique and careful intraoperative assessment of margins could allow for complete tumor removal while preserving critical structures.^5^^,^^7^

The learning curve is longer due to the rareness of the entity, we highlight some principles: correct plane dissection allows sufficient en bloc resection without interfering with the continuity of the thumb structures; if we are not certain of free margins we extend our excision even if that affects the functionality. Management should be individualized based on the complexity of vascularity and the involvement of functional units.

The limitations of this study, are based on the fact that is a case report, also the follow-up has only been for 10 months and we are still determining the long-term outcomes. We need a larger series focusing on the follow-up for objectively measuring the probability of recurrence.

## Novelty and clinical significance

This case highlights unique aspects that contribute to the literature on SCS in the hand. First, the diagnostic complexity underscores the importance of immunohistochemistry, as the initial biopsy suggested dermatofibroma, but final histopathology revealed HGSCC. Second, the surgical approach was tailored to preserve hand functionality while achieving clear margins. Despite the tumor's large size and vascular complexity, we avoided sacrificing any functional units, demonstrating the feasibility of complete preservation in carefully selected cases. Third, this case emphasizes the critical role of a multidisciplinary team in optimizing outcomes for rare sarcomas. These insights provide a framework for managing similar cases in the future.

## Conclusion

SCS in the hand underscores the need for a meticulous surgical approach that balances complete resection with the preservation of hand functionality. A thorough histopathological examination, including immunohistochemistry, is essential. Multidisciplinary collaboration, involving plastic surgery, oncology, and pathology, is crucial to optimizing patient outcomes. This case demonstrates that, even in large and complex tumors, it is possible to achieve oncological safety and functional preservation through careful planning and adaptability. Our findings contribute to the limited literature on SCS in the hand and provide a valuable framework for managing similar cases in the future.

## Declaration of competing interest

The authors declare that they have no known competing financial interests or personal relationships that could have appeared to influence the work reported in this paper.
